# Acute Thyroid Swelling After Fine-Needle Aspiration in von Willebrand Disease: Case Report and Literature Review

**DOI:** 10.7759/cureus.84532

**Published:** 2025-05-21

**Authors:** Takanobu Saheki, Toshihiro Kobayashi, Kensaku Fukunaga, Hitomi Imachi, Koji Murao

**Affiliations:** 1 Endocrinology and Metabolism, Faculty of Medicine, Kagawa University, Kagawa, JPN

**Keywords:** acute thyroid swelling, fine-needle aspiration cytology, hematoma, vasodilatory substances, von willebrand disease

## Abstract

Fine-needle aspiration cytology (FNAC) can cause rare complications, including acute transient thyroid swelling (ATTS). We report a 71-year-old female with von Willebrand disease (VWD) who developed rapid thyroid enlargement post-FNAC. Ultrasonography showed hypoechoic areas without blood flow, suggesting interstitial edema rather than hematoma. The swelling resolved within 12 hours with conservative management. Neuropeptide and histamine release may contribute to this phenomenon. This case highlights the importance of coagulation assessment and monitoring in FNAC for bleeding disorders. Recognizing hypoechoic areas without blood flow on ultrasound (US) may aid in distinguishing edema from hematoma. Further studies are needed to clarify the mechanism.

## Introduction

Fine-needle aspiration cytology (FNAC) of the thyroid gland is a pivotal diagnostic tool for differentiating benign and malignant thyroid lesions. Although this procedure is generally considered safe, complications may occur [1,2]. Acute transient thyroid swelling (ATTS) and hematoma formation have been reported, with severe cases potentially resulting in airway obstruction. ATTS occurs in approximately 0.15% of cases and is characterized by rapid enlargement of the thyroid gland immediately following the procedure, typically resolving within 1-20 hours [3]. The development of a post-aspiration hematoma is a significant clinical concern.

Von Willebrand disease (VWD), the focus of the present case, is a hereditary bleeding disorder caused by quantitative or qualitative deficiencies in the clotting factor von Willebrand factor (VWF), leading to impaired coagulation and increased bleeding risk. In Japan, the reported prevalence is approximately 0.6 cases per 100,000 individuals [4]. The management of bleeding in patients with VWD typically involves the use of desmopressin (DDAVP) or factor VIII concentrates [5,6].

This report presents a detailed account of a patient with VWD who developed ATTS following FNAC of the thyroid gland, contributing to the limited body of knowledge regarding this rare complication in patients with bleeding disorders.

## Case presentation

**Figure 1:**
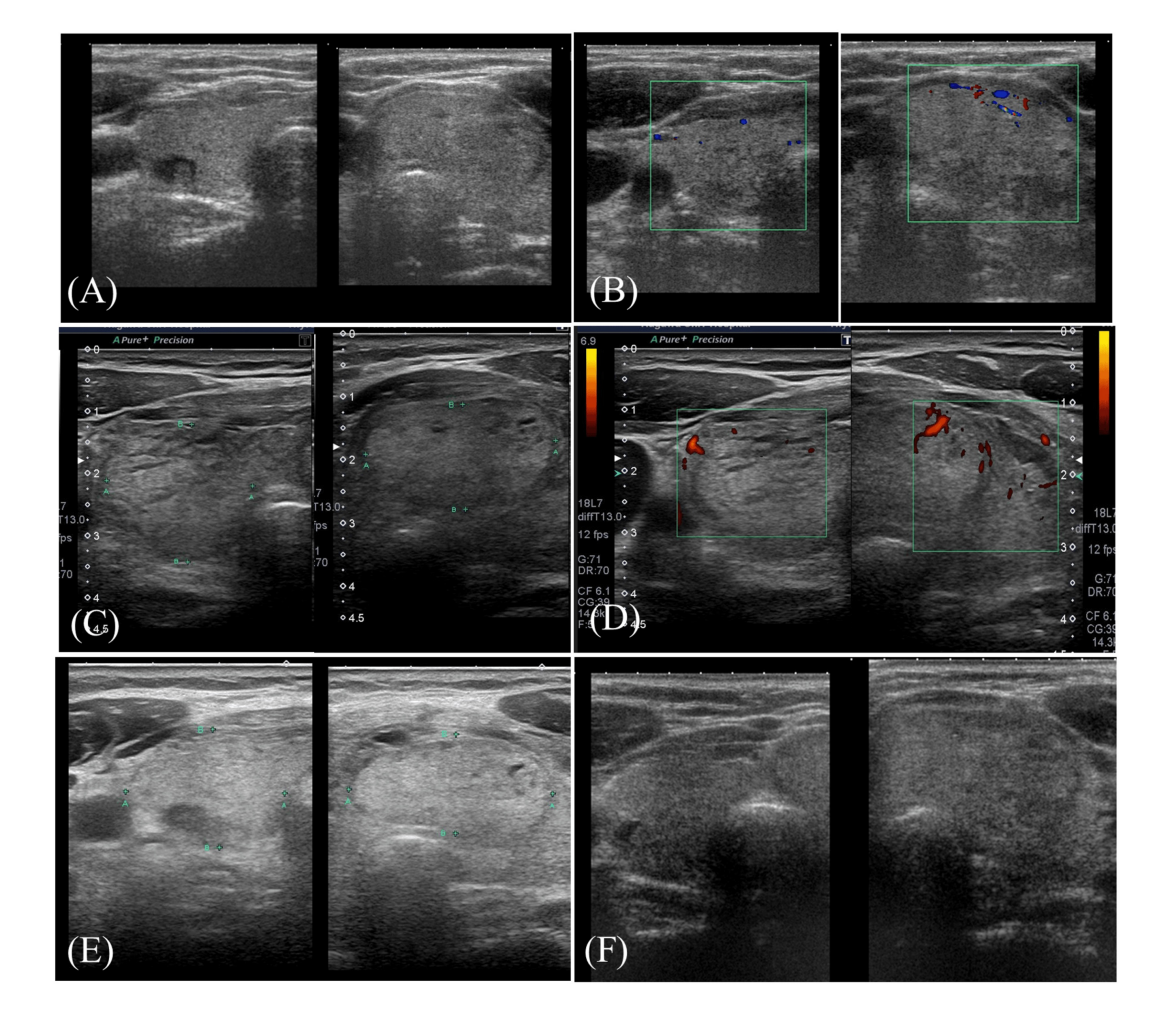
US and Doppler images of the thyroid gland before and after FNAC (A) The thyroid gland exhibits diffuse enlargement with a cystic lesion containing a solid component in the right lobe and a nodular lesion in the left lobe. (B) Doppler image corresponding to (A) shows no significant blood flow within the nodule. (C) Following FNAC of the left lobe nodule, both thyroid lobes exhibit significant enlargement with branch-like hypoechoic areas ("cracks") devoid of blood flow. Similar hypoechoic areas are also observed in the contralateral lobe. Additionally, a new hypoechoic area is visible between the thyroid gland and the muscle layer. (D) Doppler image corresponding to (C) shows unchanged vascularity around the lesion with no detectable blood flow within the hypoechoic areas. (E) Approximately 15 hours post FNAC, US reveals partial persistence of the branch-like hypoechoic areas, though marked improvement is noted. (F) One month later, the thyroid gland returned to its pre-procedural appearance, with resolution of the hypoechoic lesions in both lobes. US: Ultrasound; FNAC: Fine-needle aspiration cytology

The patient was a 71-year-old woman under observation for a thyroid tumor and attending our hematology department due to VWD, which had been diagnosed at age 29. Her medical history included an ectopic pregnancy, massive postpartum hemorrhage at the age of 26 years, hepatitis C, and dyslipidemia. The family history included atypical VWD in her oldest daughter. She had no known allergies to food or drugs and denied a history of smoking or alcohol consumption. Pre- and post-procedural ultrasonography and Doppler ultrasonography of the patient was conducted (Figure 1). A 20 mm × 16 mm lesion with signs of enlargement was identified in the left lobe of the thyroid gland, and FNAC was scheduled. The nodule was 20 mm in size and FNAC was performed due to the observed swelling and the patient's concerns. This decision was made after thorough discussion with the patient and her family, considering both the benefit and the risk of bleeding. Pre-procedural ultrasonography identified a cystic lesion in the right lobe and a nodular lesion in the left lobe (shown in Figure 1A). Pre-procedural Doppler ultrasonography showed no evident blood flow within the nodule (shown in Figure 1B). Therefore, FNAC was performed on the nodular lesion in the left lobe. FNAC was performed using a 22-gauge needle, and a single puncture was made. However, after aspiration, blood was observed in the syringe, prompting cessation of the procedure and application of compression.

Subsequently, the patient reported discomfort and pain in the neck near the puncture site and rapid enlargement of the thyroid gland was observed. Immediate ultrasound (US) revealed a branching hypoechoic area with no blood flow within the left lobe along with a newly identified hypoechoic area between the thyroid gland and muscle layer (shown in Figure 1C). Additionally, similar branching hypoechoic areas were observed in the right lobe that had not been punctured. Color Doppler imaging corresponding to Figure 1C (Figure 1D) showed no change in vascularity around the lesion and no detectable blood flow within the hypoechoic areas.

**Figure 2:**
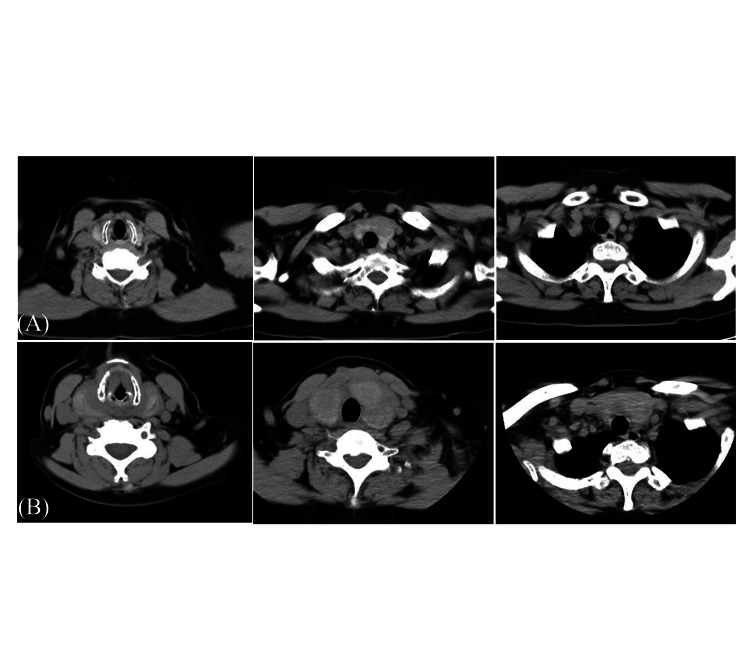
CT images obtained before (A) and after (B) FNAC (A) Pre-FNAC CT shows diffuse thyroid enlargement with an LDA within the gland. (B) Post-FNAC CT demonstrates marked enlargement of both thyroid lobes. The internal attenuation values exhibit heterogeneous reduction, and a slightly hyperattenuating soft tissue area, approximately 50 HU, is observed surrounding the thyroid. FNAC: Fine-needle aspiration cytology; LDA: Low-density area

Given rapid enlargement, neck CT was performed. Compared with previous images, marked enlargement of both thyroid lobes was observed, with heterogeneous internal attenuation and a slightly hyperattenuating soft tissue shadow of approximately 50 HU around the thyroid, suggesting the possibility of a hematoma (shown in Figure 2). At this stage, it was unclear whether the findings represented transient thyroid enlargement after FNAC or hematoma formation due to bleeding.

Assuming transient thyroid enlargement, 100 mg hydrocortisone was administered, along with neck cooling. Given the bleeding tendency associated with VWD, the patient was treated with a concentrated human coagulation factor VIII preparation and admitted to the hospital (Table 1). After treatment, the neck enlargement subsided, and by the following day, the thyroid had returned to its original size with improvement in neck discomfort (shown in Figure 1E). During the follow-up period, the patient showed no signs of purpura, ecchymosis, dyspnea, or hoarseness. 

Ultrasonography revealed a partial resolution of the branching hypoechoic areas, and the patient was discharged. A follow-up thyroid US one-month later revealed that both lobes had returned to their original appearance (shown in Figure 1F). Cytology results were returned later; however, the findings were predominantly composed of blood components, making it impossible to reach a definitive diagnosis. Cytological evaluation was inconclusive due to a predominance of blood components, and the specimen was classified as Bethesda Category I (non-diagnostic). The thyroid nodules are currently under investigation.

**Table 1:** Key laboratory findings at the time of admission are summarized Summary of the patient's key laboratory data, including complete blood count and endocrinological parameters. Reference ranges are shown for comparison. AST: Aspartate aminotransferase; ALT: Alanine aminotransferase; GGTP: Gamma-glutamyl transpeptidase; HbA1c: Hemoglobin A1c; TSH: Thyroid-stimulating hormone; FT3: Free triiodothyronine; FT4: Free thyroxine; Tg: Thyroglobulin; TPO: Thyroid peroxidase; CEA: Carcinoembryonic antigen; PT: Prothrombin time; INR: International normalized ratio; APTT: Activated partial thromboplastin time; FIB: Fibrinogen; D-D: D-dimer; VWF: von Willebrand factor; PLG: Plasminogen

Laboratory findings on admission		
Parameter	Patient Value	Reference Range (Units)
White blood cell count	4,930	4,700–8,700 /μL
Red blood cells	474	370–490 ×10⁴/μL
Hemoglobin	14.1	11.0–15.0 g/dL
Hematocrit	41.7	35.0–45.0%
Platelet count	21.5	15.0–35.0 ×10⁴/μL
Total protein	7.7	6.5–8.2 g/dL
Albumin	4.3	3.5–5.5 g/dL
Blood urea nitrogen	9.8	7.0–20.0 mg/dL
Creatinine	0.6	0.5–1.0 mg/dL
AST	22	10–35 U/L
ALT	15	5–40 U/L
GGTP	24	0–30 U/L
Sodium (Na)	142	135–146 mmol/L
Potassium (K)	4.2	3.5–4.6 mmol/L
Chloride (Cl)	108	96–110 mmol/L
Glucose	104	70–109 mg/dL
HbA1c (on admission)	5.9	4.6–6.2%
TSH	1.26	0.35–4.94 μIU/mL
FT3	3.26	1.71–3.71 pg/mL
FT4	1.22	0.70–1.48 ng/dL
Tg	83	0–33.7 ng/mL
Anti-TPO antibody	9	0–28 IU/mL
Anti-Tg antibody	14.3	0–16 IU/mL
Calcitonin	<0.5	0–3.91 pg/mL
CEA	2.6	0–5 ng/mL
PT	123	80–100%
PT-INR	0.92	0.85–1.15
APTT	32.7	24–32 s
FIB	342	200–400 mg/dL
D-D	2.1	0–1 µg/dmL
VWF	10	50–150%
PLG	102	80–130%

## Discussion

In the present case, rapid thyroid swelling developed following FNAC and resolved spontaneously within approximately 12 hours. According to the literature, > 50% of transient thyroid swelling cases manifest within 10 minutes post FNAC, whereas 36.8% occur after more than one hour [7]. Swelling frequently extends to the contralateral thyroid lobe and patients often report neck pain or discomfort. These symptoms typically subside spontaneously within 1-20 hours [3]. US examination commonly reveals fissures within the thyroid gland with no detectable blood flow in these areas, suggesting interstitial edema [8]. These US findings provide critical diagnostic insights into transient thyroid swelling [9,10]. To our knowledge, there have been no previous reports indicating that FNAC is more likely to cause interstitial edema in patients with bleeding disorders. This case may therefore offer new insights into a rare post-procedural presentation in this population. To ensure the reliability of the imaging findings, all US images were obtained using the same US machine and standardized settings, including gain and scale. The absence of blood flow in the hypoechoic areas was confirmed using Doppler imaging. These measures allowed for consistent interpretation and comparison of US findings over time.

The proposed mechanism underlying transient thyroid swelling involves stimulation of thyroidal nerve endings by the FNAC procedure, potentially triggering the release of vasodilatory substances such as substance P and neurokinin A [11]. Additionally, FNAC may induce histamine release from mast cells, further promoting neuropeptide secretion, increasing blood flow, and enhancing vascular permeability, thereby contributing to transient thyroid swelling [3].

This is the first documented case of acute thyroid swelling following FNAC in a patient with VWD. Both conditions are rare and raise the possibility of an underlying association. VWF, a multimeric protein stored in cytoplasmic granules, is released in response to stimuli such as thrombin, fibrin, or histamine [6]. Given the potential involvement of histamine in the transient thyroid swelling observed in this case, it is plausible that abnormal VWF secretion plays a role in its pathophysiology. Although the dysfunction of VWF may predispose to altered vascular responses, it remains unclear whether it increases or decreases the likelihood of transient swelling. This report underscores the need for further investigation into the relationship between VWD and transient thyroid swelling. Although it remains unclear whether VWD directly contributed to the development of transient thyroid swelling in this patient, the possibility cannot be excluded. Transient thyroid swelling can occur even in patients without bleeding disorders, and this event may have been coincidental. In addition, bleeding disorders such as VWD may increase the likelihood of obtaining nondiagnostic samples during FNAC due to increased blood contamination, which can obscure cytological findings. However, given that VWF can be released in response to histamine and other vasoactive stimuli, which are implicated in the pathogenesis of thyroid swelling, it is reasonable to hypothesize a potential interaction. Further studies and accumulation of similar cases are needed to clarify whether patients with VWD have an increased susceptibility to this rare complication. When evaluating the differential diagnosis of acute thyroid swelling after FNAC, hematoma formation due to bleeding at the puncture site should be prioritized. Hematoma-induced swelling may cause airway deviation or obstruction, which sometimes necessitates intubation. In the present case, the patient had VWD, a coagulation disorder that increased the risk of hematoma formation post FNAC. Concentrated dried human blood coagulation factor VIII was administered to mitigate this risk. However, hematoma formation was ultimately ruled out, as hematomas typically take several days to resolve [12]. This case highlights the importance of evaluating a patient's coagulation history before performing FNAC. Although discontinuation of anticoagulant or antiplatelet agents is not always necessary, prolonged compression at the puncture site and close monitoring for hematoma formation are advised. No other potential causes of post-FNAC thyroid swelling, such as allergic reactions to the needle material, disinfectants, or US gel, were observed [13].

Additionally, the timing of thyroid swelling onset may help differentiate the underlying etiology. ATTS has been reported in patients with follicular carcinoma, follicular adenoma (FA), papillary carcinoma (PC), medullary carcinoma, and adenomatous goiters [8,14]. In this case, the pathological evaluation was inconclusive owing to blood contamination of the sample.

This case highlights the challenges in predicting transient thyroid swelling following FNAC and the critical importance of thorough pre-procedural assessment, early diagnosis, and appropriate management. A comparative summary of previously reported cases of ATTS following FNAC, including the present case, is provided in Table 2. This table highlights similarities and differences in onset time, US features, treatment strategies, and clinical course. Although transient thyroid swelling typically resolves spontaneously without specific intervention, its rapid progression necessitates vigilant monitoring. US findings are instrumental in the diagnosis and management of patients with VWD presenting with bleeding tendencies, as demonstrated in this case. These imaging findings also provide valuable guidance for the clinical management of transient thyroid swelling following FNAC.

**Table 2:** Summary of previously reported cases of acute thyroid swelling following fine-needle aspiration Comparative summary of previously reported cases of ATTS following FNAB, including the present case. The table highlights differences in demographics, clinical features, US findings, treatment, and resolution. ATTS: Acute transient thyroid swelling; FNAB: Fine-needle aspiration biopsy; FA: Follicular adenoma; PC: Papillary carcinoma; VWD: Von Willebrand disease; US: Ultrasound

Comparative Summary of ATTS Cases Following FNAB								
Study	Age/Sex	Nodule Type	Onset Time	US Findings	Treatment	Resolution Time	Notes	
Watanabe et al. [3]	40/F	Follicular tumor	~30 min	Crack-like hypoechoic areas, bilateral, no flow	Steroid IV + oral	~18 h (dysphagia remained for 72 h)	Swelling extended to perithyroid muscles	
Yoshimoto et al. [9]	27–82/M&F	Various (adenoma, goiter, carcinoma)	Immediate to 2 h	Hypoechoic cracks, unilateral/bilateral	Cooling, steroids in some	1–14 days (many within hours)	Large retrospective series (n=41)	
Mizokami et al.[12]	31–72/M&F	Multinodular goiter, FA, PC	Immediate to 2 h	Hypoechoic cracks, bilateral	Steroid IV or oral / Cooling	Within hours to few days	Prevalence: 0.10% (10/9596 nodules)	
Present Case	71/F	Cystic and solid nodule	~10 min	Crack-like hypoechoic areas, bilateral, no flow	Steroid IV + factor VIII + cooling	~12 h	Only reported case with VWD	

## Conclusions

This case highlights a rare but significant complication of FNAC in a patient with VWD. The rapid onset and resolution of bilateral thyroid swelling, along with characteristic hypoechoic cracks on ultrasonography without blood flow, strongly suggested interstitial edema rather than hematoma. Although a causal relationship between VWD and this phenomenon remains speculative, the involvement of histamine and vasoactive substances provides a plausible mechanism. This case emphasizes the importance of thorough coagulation assessment and vigilant monitoring when performing FNAC in patients with bleeding disorders. Prompt imaging evaluation and conservative management led to a favorable outcome in our case. Accumulation of further cases will help clarify the pathophysiological association between VWD and transient thyroid swelling.
